# Effect of Baseline BMI and IL-6 on Gait Speed Response to Caloric Restriction in Older Adults

**DOI:** 10.1093/geroni/igab046.302

**Published:** 2021-12-17

**Authors:** Katherine Hsieh, Rebecca Neiberg, Kristen Beavers, Daniel Beavers

**Affiliations:** 1 Wake Forest School of Medicine, Winston Salem, North Carolina, United States; 2 Wake Forest University, Winston Salem, North Carolina, United States; 3 Wake Forest School of Medicine, Winston-Salem, North Carolina, United States

## Abstract

We examined whether the effect of caloric restriction (CR) on gait speed change in older adults (67.3±5.27 years) varied by BMI and interleukin 6 (IL-6). Data from eight six-month randomized controlled trials were pooled, with 1268 participants randomized to CR (n=710) and non-CR (n=558) conditions. Baseline BMI/IL-6 subgroups were constructed using BMI≥35 kg/m2 and IL-6>2.5 pg/dL, and participants were jointly classified as high/high (n=395), high/low (n=208), low/high (n=271), or low/low (n=344). Overall treatment effects showed significant improvements in gait speed in CR versus non-CR [mean difference: 0.02 m/s (95% CI: 0.01, 0.04)]; however, CR assignment significantly interacted with BMI/IL-6 subgroup (p=0.03). Greatest gait speed improvement was observed in the high/high CR subgroup [+0.06 m/s (0.03, 0.09)] and appeared to be driven by no gait speed change among the high/high non-CR subgroup. Gait speed response to CR was greatest in older adults with elevated baseline BMI and IL-6.

